# Osteo-immunological impact of radon spa treatment: due to radon or spa alone? Results from the prospective, thermal bath placebo-controlled RAD-ON02 trial

**DOI:** 10.3389/fimmu.2023.1284609

**Published:** 2024-01-16

**Authors:** Denise Eckert, Megi Evic, Jasmin Schang, Maike Isbruch, Melissa Er, Lea Dörrschuck, Felicitas Rapp, Anna-Jasmina Donaubauer, Udo S. Gaipl, Benjamin Frey, Claudia Fournier

**Affiliations:** ^1^ Department of Biophysics, GSI Helmholtzzentrum Für Schwerionenforschung, Darmstadt, Germany; ^2^ Translational Radiobiology, Department of Radiation Oncology, Universitätsklinikum Erlangen, Friedrich-Alexander-Universität Erlangen-Nürnberg (FAU), Erlangen, Germany

**Keywords:** osteoclastogenesis, radon and thermal spa treatment, bone metabolism, Th17/Treg cells, biomarker and degenerative musculoskeletal diseases

## Abstract

Musculoskeletal disorders (MSDs) are associated with pain and lead to reduced mobility and quality of life for patients. Radon therapy is used as alternative or complementary to pharmaceutical treatments. According to previous reports, radon spa leads to analgesic and anti-inflammatory effects, but the cellular and molecular mechanisms are widely unknown. A previous study (RAD-ON01) revealed, that bone erosion markers like collagen fragments (C-terminal telopeptide, CTX) are reduced after radon spa treatment in serum of patients with degenerative MSDs. Within the scope of the prospective, placebo-controlled RAD-ON02 trial presented here, we analyzed the influence of radon and thermal spa treatment on osteoclastogenesis. From patient blood, we isolate monocytes, seeded them on bone slices and differentiated them in the presence of growth factors into mature osteoclasts (mOCs). Subsequent analysis showed a smaller fraction of mOCs after both treatments, which was even smaller after radon spa treatment. A significantly reduced resorbed area on bone slices reflects this result. Only after radon spa treatment, we detected in the serum of patients a significant decrease of receptor activator of NF-κB ligand (RANKL), which indicates reduced differentiation of OCs. However, other markers for bone resorption (CTX) and bone formation (OPG, OCN) were not altered after both treatments. Adipokines, such as visfatin and leptin that play a role in some MSD-types by affecting osteoclastogenesis, were not changed after both treatments. Further, also immune cells have an influence on osteoclastogenesis, by inhibiting and promoting terminal differentiation and activation of OCs, respectively. After radon treatment, the fraction of Treg cells was significantly increased, whereas Th17 cells were not altered. Overall, we observed that both treatments had an influence on osteoclastogenesis and bone resorption. Moreover, radon spa treatment affected the Treg cell population as well as the Th17/Treg ratio were affected, pointing toward a contribution of the immune system after radon spa. These data obtained from patients enrolled in the RAD-ON02 trial indicate that radon is not alone responsible for the effects on bone metabolism, even though they are more pronounced after radon compared to thermal spa treatment.

## 1 Introduction

Musculoskeletal disorders (MSDs) include degenerative and inflammatory diseases, such as Rheumatoid arthritis (RA), Osteoarthritis (OA) or Calcaneodynia. Bone degradation and/or destruction of joints structure are hallmarks of MSDs (1, 2). This leads to pain, the main symptom of MSDs, and to inflammation, stiffness and joint swelling, severely reducing physical function and health-related quality of life.

Standard pharmaceutical treatment consists in administration of non-steroidal anti-inflammatory drugs (NSAID)/non-steroidal antirheumatics (NSARs) or biologicals. However, these treatments are associated with high costs and have negative side effects, such as gastrointestinal bleeding, myocardial infarction, and stroke (3–5). Thus, there is a huge demand for alternative or complementary, well-tolerated treatments, such as low-dose photon radiation therapy (LDRT) or radon therapy (via galleries or spa). For LDRT, patients are treated with a total dose of 3-6 Gy, which is administered locally in fractionated doses of 0.5 Gy in 6-12 treatment sessions (6). Radon spa treatment consists of repeated exposures in baths or in galleries, with an estimated range of activity concentrations of 0.3-3 kBq/l for a radon bath (20 min bathing time), or 30-160 kBq/m^3^ in galleries (1 hour visit) (7). After both treatment modalities (LDRT and radon treatment), the patients benefit from long-lasting pain reduction, amelioration of inflammatory processes and immune modulation (7–14). So far, the underlying mechanisms are not entirely understood, but an influence on the immune system and bone metabolism was anticipated based on the results of the observatory RAD-ON01 trial (9, 15). In brief, the prospective and exploratory RAD-ON01 study found that collagen fragment (CTX) decreased significantly, suggesting decreased bone resorption. Furthermore, we observed an increase of regulatory T cells in the peripheral blood and a reduced level of visfatin, indicating immune suppression and a reduction of inflammation (15). As a next step, a prospective, double-blinded and placebo-controlled follow-up study (RAD-ON02) was initiated to exclude a placebo effect. Part of the results are presented here.

In healthy tissue, bone is undergoing continuous remodeling, especially by bone-forming osteoblasts (OBs), osteocytes and resorbing osteoclasts (OCs) (16). For MSDs, a disturbed balance between active OCs and OBs by an increase in the proportion of actively resorbing OCs results in increased bone resorption. In this study (RAD-ON02), we focus on patients suffering from degenerative MSDs, and investigate the effect of radon and thermal spa treatment on bone metabolism, in particular on the bone resorption activity of terminal differentiated OC.

Mature OCs (mOCs) are multinucleated bone-resorbing cells derived from the monocyte/macrophage lineage by fusion of several precursor cells. The cytokines macrophage colony-stimulating factor (M-CSF) and receptor activator of NF-κB ligand (RANKL) are essential for the onset of differentiation. First, monocytes differentiate into pro-osteoclasts (pro-OCs) under the influence of M-CSF (17). RANKL, a member of the TNF family expressed on the surface of OBs/stromal cells, binds to receptor activator of NF-κB (RANK) on pro-OCs, causing them to further differentiate into pre-OCs. Fusion of pre-OCs eventually gives rise to multinucleated (≥ 3 nuclei) bone-resorbing mOCs.

An antagonist of RANKL is osteoprotegerin (OPG), a soluble decoy receptor released by osteoblasts. OPG binds RANKL and thus inhibits osteoclastogenesis, and is as such considered as a marker for bone formation (18). Mature OCs migrate to bone where bone resorption is detectable by the formation of resorption pits and release of collagen fragments (such as CTX). Tartrate Resistant Acid Phosphatase (TRAP) 5b activity and three or more nuclei in one cell are established markers for bone resorbing mOCs. The close cell-matrix contact is mediated by integrins - in particular, integrin αvβ3 (19). This leads to the formation of an F-actin ring that defines the area to be resorbed by sealing (sealing zone); the presence of an F-actin ring is also a marker for actively resorbing mOCs. In addition to RANKL and CTX (bone resorption) and OPG (bone formation), osteocalcin (OCN) is also a typical molecule involved in bone formation. OCN is the most abundant non-collagenous protein in bone and is synthesized by OBs, odontoblasts, and hypertrophic chondrocytes (20, 21). An increase in OPG and OCN, as well as a decrease of RANKL and CTX release is considered an indication of an anabolic influence on bone metabolism, which we investigated in the present work for radon spa treatment in patient serum before, during and after treatment.

Immune cells, such as regulatory T cells (Treg) cells and T helper 17 (Th17) cells have an influence on osteoclastogenesis. Treg cells may have immune suppressive effects (22). In addition, by preventing the production of M-CSF and RANKL, they can inhibit the terminal differentiation and activation of OCs, leading to an increase in bone mass (23). In contrast, Th17 cells have inflammatory effects, and by expressing high surface levels of RANKL, which binds to RANK on the surface of OC progenitor cells, they can promote the terminal differentiation of osteoclast progenitor cells into OCs, leading to increased bone resorption. Th17 cells also enhance the expression of RANKL in OBs and synovial fibroblasts by the release of IL-17 (24). We measured fractions of relevant T cells in patient blood during and after radon spa treatment in this study.

Beside bone erosion, progressive destruction of articular cartilage is e.g. a characteristic of OA (25). Here, extracellular matrix (ECM)-degrading enzymes such as matrix metalloproteinases (MMPs) play a major role. MMPs are zinc-containing endopeptidases that are increased in expression in response to excessive mechanical stress and proinflammatory cytokines (TNF-α, IL-1ß, IL-6) (21). MMP-3, also known as stromelysin-1, is released by chondrocytes and synovial cells and degrades a variety of ECM components (e.g., collagen types, fibronectin, laminin) (26). In addition, MMP-3 activates pro-MMP-9 (gelatinase B), which is produced by OCs and may therefore be involved in bone resorption (27, 28). This idea is supported by the fact that cathepsin K (derived from osteoclasts) can activate proMMP-9 under acidic conditions (29). In our study, we investigated in the serum of patients the influence of radon and thermal spa treatment on the level of these markers of cartilage destruction, i.e. MMP3 and MMP9.

In OA patients, the systemic levels of the adipokines visfatin and leptin are elevated, both produced mainly by adipocytes (30, 31). Inhibiting adipogenic differentiation in the bone marrow, leptin leads to an increase in proliferation and differentiation of OBs. By inducing collagen synthesis, bone mineralization, OB proliferation and differentiation as well as endochondral ossification, leptin leads to bone growth (32–34). In contrast, visfatin fosters bone and cartilage destruction by inhibiting ECM formation via reduction of the production of proteoglycans and collagen type II (35). In addition, mice treated with visfatin showed increased expression of MMP3, MMP-13, ADAMTS-4, and ADAMTS-5, which leads to degradation of ECM and aggrecans (36). Since it is known that radon accumulates better in fat than in water (37), the investigation of the influence by radon on the level of adipokines is of major relevance.

In this study, we aimed to investigate whether the long-lasting pain reduction and lowered expression of bone erosion markers that were observed in our previous study (8, 15) were due to radon alone or due to a spa effect. For this purpose, a prospective, double-blinded and temporary placebo-controlled trial (RAD-ON02), which is a follow-up study of the RAD-ON01, was initiated using a cross-over design. In the work presented here, we assessed osteoimmunological effects using blood drawn from radon versus thermal bath exposed patients. We quantified *ex vivo* the fraction of terminally differentiated OCs, OC precursors and the resorbed area on bone slices. In addition, we examined the influence of both treatments on a systemic level by quantifying adipokines (visfatin and leptin), markers of bone resorption (CTX, RANKL) and formation (OPG, OCN) as well as markers of cartilage destruction (MMP3, MMP9). Furthermore, we investigated the ratio of immune promoting and inhibiting T cells (Th17/Treg ratio) which influence bone formation and resorption and expected a decreased Th17/Treg ratio in the blood of the patients.

## 2 Materials and methods

### 2.1 Study design of the RAD-ON02 trial

The RAD-ON02 study is a prospective, double-blinded and placebo-controlled trial (EudraCT Nr. 2016-002085-31, DRKS-ID DRKS00016019). This is the follow-up study of the RAD-ON01 study, which had no control group (8, 15). A total of 116 patients suffering from musculoskeletal and chronic degenerative disorders of the spine and joints. were included in the RAD-ON02 study. Informed consent was obtained from all patients. The trial was ethically approved by the Institutional Review Board of the Bayerische Landesärztekammer (BLÄK) in 2017 and followed the ‘Declaration of Helsinki’ in its current form. Patients with age at least 18 years, up to 75 years, chronic degenerative spinal and joint complaints, duration of pain for at least 1 year and pain intensity (visual analog scale) VAS ≥4 were included in the study. The detailed inclusion and exclusion criteria are listed in **Supplementary Table 1**.

**Figure 1:**
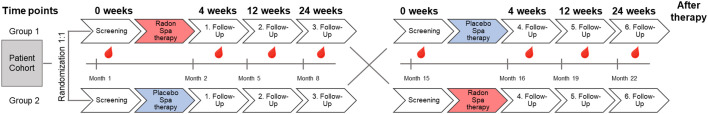
Study design of the prospective, double-blind and temporary placebo-controlled RAD-ON02 trial.

The study was carried out according to a cross-over design. Patients were randomized in two cohorts in a double-blinded manner; neither the treating physicians or the involved scientists nor the patient knew which treatment was performed. In the first year, one group first received thermal spa treatment while the other group received radon spa treatment with a radon concentration of 1200 bq/L at the state-certified health resort Bad Steben (Germany). Both groups received nine baths for 20 min in a period of three weeks. Before treatment (0 weeks), pain-related data were collected and blood was drawn from both groups. This was repeated in week 4 directly after the bath series as well as 12 and 24 weeks after the treatment. One year after the first bath treatment, the treatment was changed for both cohorts, so that the cohort that first received the radon spa treatment then received the thermal spa treatment and vice versa for the other cohort. Again, pain-related data were collected and blood was drawn the week before (0 weeks) and directly after (week 4) as well as 12 and 24 weeks after the second bath series (**Figure 1**).

In this work, subgroups of patients have been randomly analyzed. Characteristics of these patients are shown in **Table 1**. The detailed split of the patients into the two groups is shown in **Supplementary Table 2**. For *ex vivo* investigation of osteoclastogenesis, Peripheral Blood Mononuclear Cells (PBMCs) were isolated from patients’ blood (see 2.2). PBMCs were differentiated into mOCs on bone slices in the presence of growth factors (RANKL and M-CSF) and the total number of OCs, fraction of mOCs, and resorbed area were quantified (see 2.2, 2.3). In addition, the fraction of Treg and Th17 cells were determined in the PBMCs and related to lymphocytes, according to Eckert et al. (see 2.5) (38). Furthermore, the concentration of markers related to bone metabolism and inflammatory key players, such as adipokines, were investigated in serum or plasma of the patients before and at different time points after treatment. We received only a subset of patients and from these we do not have blood samples from all time points, therefore the number of patients in the experiments differs. In addition, the number of isolated PBMCs was not always sufficient to perform all experiments.

**Table 1:** Characteristics of patients (RAD-ON02 trial).

Total number		58	patients
Age at start	Mean	60	years
	Range	40-73	years
Gender	Male	20 (34.5%)	patients
	Female	38 (65.5%)	patients
BMI	Normal (<25)	14 (21.1%)	patients
	Overweight (25-30)	32 (55.2%)	patients
	Obese (>30)	12 (20.7%)	patients
Indications	Joints	3 (5.2%)	patients
	Spine	6 (10.3%)	patients
	Multiple Indications	49 (84.5%)	patients

### 2.2 Isolation and cultivation of OC precursors and differentiation into mOCs

For the isolation of human PBMCs, blood from patients was collected in vacutainer tubes (BD vacutainer^®^ blood collection tubes, BD Biosciences, Heidelberg, Germany at the state-certified health resort in Bad Steben, and was shipped to GSI Helmholtzzentrum für Schwerionenforschung (Darmstadt, Germany) as described by Eckert et al. (38). Briefly, the next day, vacutainer tubes were centrifuged at 1500 xg for 20 min at room temperature (RT). For cultivation, plasma was removed and heat-inactivated (30 min at 56°C). Denatured proteins were removed (3360 xg for 5 min). PBMC were collected and washed with PBS/2% FBS (Sigma Aldrich) (300 xg, 8 min). A red blood cell lysis buffer (RBC, 8.29 g NH_4_Cl, 1 g KHCO_3_, 37.2 mg Na_2_EDTA, 800 ml H_2_O, pH 7.2-7.4 in 1 l H20) was added and incubated at RT for 5 min to remove the remaining erythrocytes. To stop the reaction, PBS/2% FBS was added. Next, cells were collected (300 xg for 8 min) and suspended in X-Vivo-15 medium (Lonza/Biozym Scientific GmbH, Oldendorf, Germany) containing 1% penicillin-streptomycin (Pen/Strep, PAN-BIOTECH, Aidenbach, Germany) and 3% autologous plasma.

Directly after cell preparation, 4x10^5^ cells were seeded on three bone slices (ids immunodiagnostics, Boldon, UK) in X-Vivo-15 medium and incubated for 1-2 hours at 37°C/5% CO_2_ in 96-well plates. After attachment, non-adherent lymphocytes were removed. The remaining attached cells on bone slices were transferred to 24-well plates, containing 500 µl of alpha-medium (Merck Millipore) supplemented with 1% Pen/Strep, 10% autologous plasma and differentiation factors, i.e., RANK-L (40 ng/ml, EMD Millipore Corp., Billerica, MA, USA) and M-CSF (25 ng/ml, Miltenyi Biotec, Bergisch Gladbach, Germany). For the following differentiation process into OCs, cells were cultured for 21 days and the medium was changed twice a week.

### 2.3 Identification of OC precursors and mOC by fluorescent triple staining

Fluorescent triple staining of OC precursors and mOCs was performed as previously described (38). Briefly, cells were fixed with 3.7% PFA (Merck KGaA, Darmstadt, Germany) (15 min at RT). Afterwards, cells were incubated with TRAP staining solution [TRAP buffer (0.1 M acetate buffer: 35.2 ml 0.2 M sodium acetate solution, 14.8 ml 0.2 M acetic acid, 50 ml; Millipore, Burlington, USA) mixed with 0.3 M sodium tartrate (pH 5.5), 10 mg/ml naphthol AS-MX phosphate, Triton X-100, 0.3 mg/ml fast red violet LB Salt (all from Sigma Aldrich Chemie GmbH)] at 37°C for 10 min. After washing, cells were stained with FITC-phalloidin (0.66 µg/ml in PBS, Sigma Aldrich Chemie GmbH) (25 min in the dark at RT). Subsequently, DAPI (1 μg/ml in PBS, BD Biosciences) was added directly and incubated for another 7 min. Cells were washed and stored at 4°C in PBS in the dark.

Using a confocal microscope (DMI4000B, Leica, Wetzlar, Germany), microscopic pictures were taken (5-10 images per bone slice). According to established criteria, the cells were classified into three groups: pro- (spindle-shaped with a single nucleus), pre- (round with one or two nuclei) and mOCs (TRAP-positive and with three or more nuclei) (39–41). Throughout the text, pro-and pre-OCs are not distinguished and termed OC precursor. ImageJ (Wayne Rasband, NIH, Bethesda, USA) was used for evaluation. The mean count of OCs and OC precursors were calculated from the evaluation of ten images.

### 2.4 Analysis of bone resorbing activity

The bone resorbing activity was visualized by staining with toluidine blue as described elsewhere (38). For this, cells were removed from the bone slices by incubation with ammonium hydroxide solution (0.25 M ammonium hydroxide solution, Sigma Aldrich Chemie GmbH, diluted with dist. water). Then, toluidine blue staining solution [1% toluidine blue (Fluka/Sigma Aldrich Chemie GmbH) solved in 1% sodium tetraborate (40°C, soluble in H_2_O; Sigma Aldrich Chemie GmbH)] was added to the bone slices for 10 min. Toluidine blue staining solution was washed away with PBS. With a fluorescence microscope (Revolve 4M, Echo, San Diego, CA, USA) six images per bone slices were taken. The colour threshold function of ImageJ was used for the analysis of the resorbed area on bone slices. If the determination of the resorbed area using the colour threshold was not possible (e.g. weak staining), the ROI (region of interest) Manager was used. The mean value was calculated from six images.

### 2.5 Analysis of Th17/Treg by Flow cytometry

**Figure 2:**
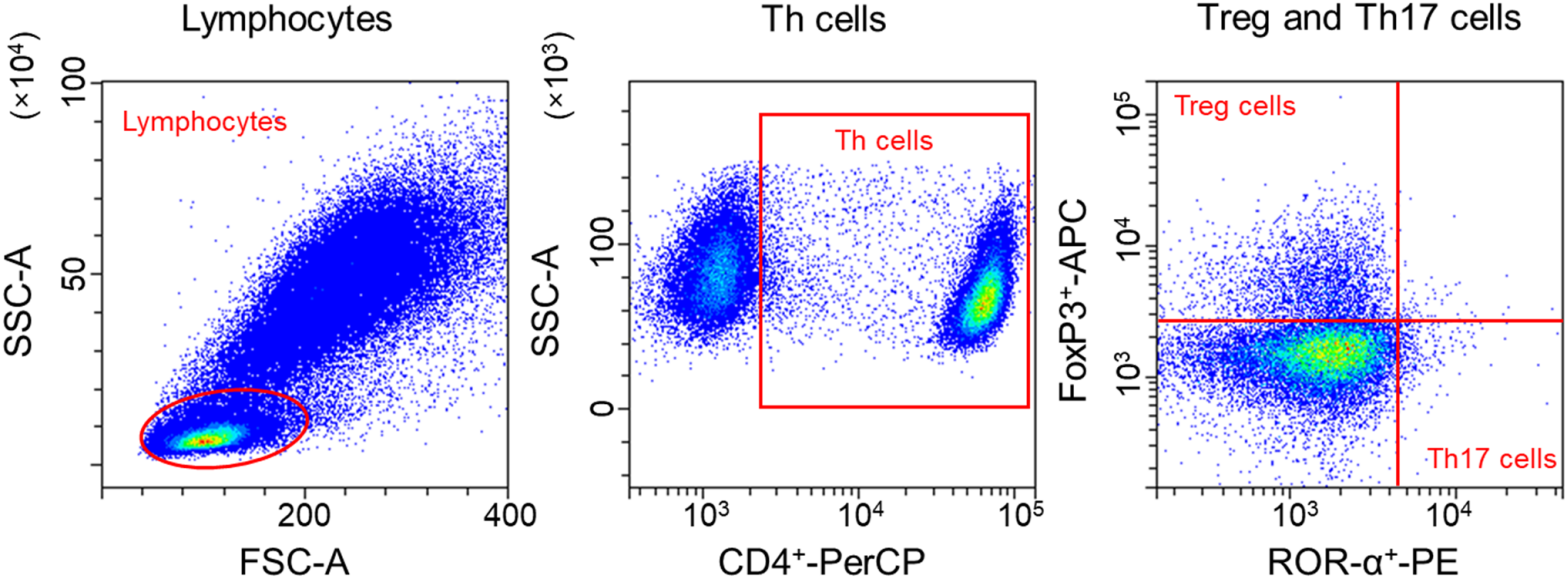
Gating strategy of Treg and Th17 cells. In the SSC/FSC dot plot, lymphocytes were determined. After exclusion of doublets, T helper cells (Th cells) (CD4^+^-Per-CP) were identified in the SSC/CD4^+^-Per-CP dot plot. Subsequently, Th17 cells (ROR-α^+^-PE) cells and Treg cells (FoxP3^+^-APC) respectively from CD4^+^ were classified.

Isolated PBMC from RAD-ON02 patients (see 2.2) were used for the analysis of Th17 and Treg cell populations as previously described (38). In brief, 1x10^6^ PBMC were fixed with 1× Human FoxP3 Buffer A (BD PharmingenTM, BD Pharmingen, Heidelberg, Germany) at RT for 20 min in the dark. Subsequently, cells were washed (500 xg for 5 min), and permeabilized with 0.5 ml of 1× working dilution of Human FoxP3 Buffer C (BD PharmingenTM) (30 min at RT in the dark). Then, cells were washed again (500 xg, 5 min). Finally, cells were stained with CD4 (PerCP-Cy5.5, clone SK3, #566923, BD), ROR-α (PE, clone # 784652, #IC8924P-100, R&D Systems, Minneapolis, USA) and FoxP3 (Alexa Fluor 647, clone 259D/C7, #560045, BD) for 40 min at RT in the dark and washed (500 xg, 5 min). The expression of intracellular and cell surface markers was measured using a flow cytometer (CytoFLEX S, Beckman Coulter, Krefeld, Germany). In the SSC/FSC dot plot, lymphocytes were determined. After exclusion of doublets, T helper cells (Th cells) (CD4^+^-Per-CP) were identified in the SSC/CD4^+^-Per-CP dot plot. Subsequently, Th17 cells (ROR-α ^+^-PE) cells and Treg cells (FoxP3^+^-APC) respectively from CD4^+^ were classified (**Figure 2**). Using CytExpert software (Beckman Coulter), the frequency of cells related to the total number of CD4^+^ cells was analyzed: CD4^+^ ROR-α+ as Th17 cells and CD4^+^FoxP3^+^ cells were classified as Treg cells. The mean value was calculated from the technical duplicates.

### 2.6 Detection of CTX fragments and OCN in plasma of patients

CTX ELISA (Serum CrossLaps^®^ (CTX-I) ELISAs, ids immunodiagnostics, Boldon, UK) and osteocalcin human ELISA Kit (Thermo Fisher, Waltham, MA, USA) were performed with plasma from patients, according to the manufacturer’s protocol, for the analysis of released fragments of collagen type I. With SpectraMax i3x (Molecular Devices, California, USA) the absorbance was measured at 450 nm with reference at 650 nm. Afterwards, the mean value was calculated from the technical duplicates.

### 2.7 Detection of TRAP 5b activity and Visfatin in serum of patients

BoneTRAP^®^ ELISA (ids immunodiagnostics, Boldon, UK) and Nampt (visfatin/PBEF) (human) ELISA Kit (AdipoGen (Liestal, Switzerland) were used for analysis of TRAP 5b activity in serum of patients, as described in the manufacturer’s protocol. With SpectraMax i3x (Molecular Devices, California, USA) the absorbance was measured at 450 nm with reference at 650 nm. Afterwards, the mean value was calculated from the technical duplicates.

### 2.8 Quantification of biomarkers for bone resorption, bone formation and adipokines using Meso Scale Discovery^®^

To analyze the biomarkers (RANKL, leptin, MMP3, MMP9 and OPG), electrochemiluminescence-based assays were performed in serum of patients using the Meso Scale Discovery^®^ (MSD^®^; Rockville, MD, USA) system according to the manufacturer’s protocol. For RANKL U-PLEX^®^ Immuno-Oncology Group 1 (human) Assay and for Leptin U-PLEX Immuno-Oncology Group 1 (human) Assay were used. For MMP3, MMP9 and OPG R-PLEX^®^ (human) Assays were applied. For detection of RANKL, MMP3, and MMP9 serum from patients were diluted 1:10 in Diluent 58. Using a MESO QuickPlex SQ120 instrument, the intensity of emitted light was measured to provide quantitative measures of analytes in samples. Data analyses were performed using MSD^®^ Discovery Workbench^®^. Afterwards, the mean value was calculated from the technical duplicates.

### 2.9 Statistical analysis and graph settings

Data and statistical analyses were calculated with GraphPad Prism 9 (GraphPad Software, Inc., La Jolla, CA, USA) and are displayed as mean values ± SEM. Prior to performing the Shapiro-Wilk test, data were tested for normal distribution. For analysis of more than two groups that are unpaired the following tests were applied: 1. for normal distributed data one-way-ANOVA followed by a posthoc Tukey’s multiple comparison test, and 2. for non-normal distributed data Kruskal-Wallis test followed by Dunn’s test. For analysis of more than two groups that are paired the following tests were used: 1. for normal distributed data repeated measures one-way ANOVA (RM one-way ANOVA) followed by posthoc Tukey’s multiple comparison tests and 2. for non-normal distributed data Friedman test. A p-value ≤ 0.05 was considered as significant.

To investigate whether the treatment of the first series has an effect on the outcome after cross-over (after tp5) (see **Figure 1**), the Carry-Over effect was calculated by using the following (**Equations 1** and **2**):


$$2\hat{\text{τ}}=\left({\bar{\text{Y}}}_{1.1}-{\bar{\text{Y}}}_{1.2}\right)-\left({\bar{\text{Y}}}_{2.1}-{\bar{\text{Y}}}_{2.2}\right)={\text{m}}_{1}-{\text{m}}_{2}$$

*m_i_* is the sum of the mean values over both periods in group i.


Equation 1 $${\text{S}}_{\text{m}}=\;\sqrt{\frac{\left({\text{n}}_{1}-1\right){\text{S}}_{1}^{2}+\left({\text{n}}_{2}-1\right){\text{S}}_{2}^{2}}{{\text{n}}_{1}+{\text{n}}_{2}-2}}$$

*n_i_* is the sample size of Group i and $S_{i}^{2}$ is the empirical variance over both periods in group i.


Equation 2 $${\text{T}}_{\text{W}}=\;\sqrt{\frac{{\text{n}}_{1}\times {\text{n}}_{2}}{{\text{n}}_{1}+{\text{n}}_{2}}}\;\times \frac{\left|{\text{m}}_{1}-{\text{m}}_{2}\right|}{{\text{S}}_{\text{m}}}$$

*m_i_ = Sum of the mean values across both periods;* $S_{\text{i}}^{2}$ *= is the empirical variance of the observation sum over both persons in group i; n_i_ = the number of cases in group i; i takes two values here: 1 and 2, and identifies the two groups;* $2\;\hat{\tau }$ *: interaction effect; S_m_*: *empirical variance; T_W_: test statistic.*

If the p-value is higher than 0.05, there is no influence of the treatment in the first year on the second treatment one year later (after cross-over) and the data of the respective therapies were pooled (42). This was applied to ELISA data (3.2, 3.3, 3.4). For the *ex vivo* analyses, only the time points before cross-over were evaluated (see 3.1, 3.5).

## 3 Results

### 3.1 Radon spa and thermal spa treatment significantly decrease osteoclastogenesis and resorbed area on bone slices

Radon spa treatment shows significantly reduced pain and a systemic decrease of markers related to bone erosion in the blood and serum of radon exposed MSDs patients in previous studies (8, 15). To investigate a potential impact of radon exposure on osteoclastogenesis, we examined the effect of radon versus thermal spa treatment in order to discriminate observed effects originating from radon alone and those caused by other factors, i.e. the exposure to warm water alone.

For this purpose, patients’ blood was collected at different time points (before (0), 4, 12 and 24 weeks) (see **Figure 1**), monocytes were isolated and *ex vivo* differentiated on bone slices in the presence of growth and differentiation factors (M-CSF, RANKL) into OCs. After cultivation for 21 days, the differentiation of monocytes into OCs was assessed by quantifying the total number of OCs, the respective fractions of mOCs and OC precursor cells. In addition, the resorbed area on the bone slices was measured.

**Figure 3:**
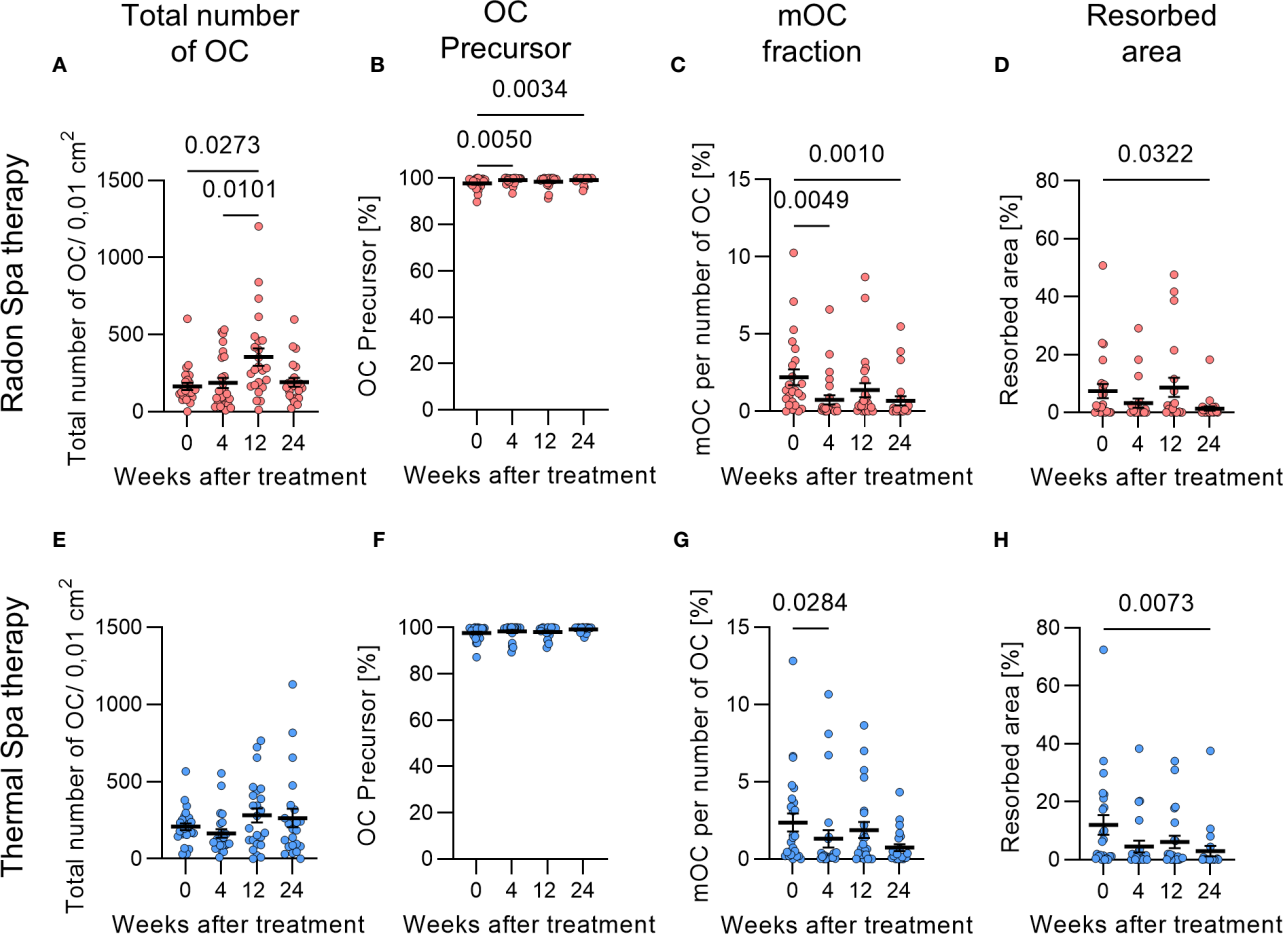
Reduced terminal differentiation and activity of bone resorbing osteoclasts (OCs) after radon and thermal spa treatment. The total number of OCs was significantly increased after radon spa **(A)** and slightly increased after thermal spa treatment **(B)** (N=22-27). The fraction of OC precursors was increased after radon spa **(C)** and thermal spa treatment **(D)** (N=24-26). The fraction of mOCs was significantly decreased after both treatments, with a higher level of significance after radon **(E)** compared to thermal spa treatment **(F)** (N=24-26). As a result, the resorbed area was also significantly reduced after radon **(G)** and thermal spa treatment **(H)** (N=21-24). Significances were tested with Kruskal-Wallis test. Error bars are reported as mean ± SEM.

After radon spa treatment (**Figure 3A**), the total number of OC/0.01cm^2^ was first significantly decreased (2-3-fold) at 12 weeks after treatment. In contrast, after thermal spa treatment the total number of OCs was not significantly increased 12 weeks after treatment in comparison to the initial time point (**Figure 3B**).

Notably, for both, radon and thermal spa treatment, a modified differentiation was observed, but the effect after radon spa treatment on the differentiation into mOCs was more pronounced and at a higher level of significance compared to thermal spa treatment, in particular for 4 and 24 weeks after radon spa treatment (**Figures 3C, D**). Correspondingly, the fractions of precursor cells after radon as well as thermal spa treatment were higher compared to before therapy (**Figures 3E, F**). The smaller fractions of mOCs observed after both treatments were reflected in smaller areas that were resorbed on bone slices. Compared to before treatment, the percentage of resorbed area on bone slices was significantly reduced by 5-fold 24 weeks after radon spa treatment (**Figure 3G**) and by 4-fold after thermal spa treatment (**Figure 3H**). The reduction after thermal spa treatment was at a higher level of significance than after radon spa treatment.

### 3.2 RANKL is significantly decreased after radon spa treatment

**Figure 4:**
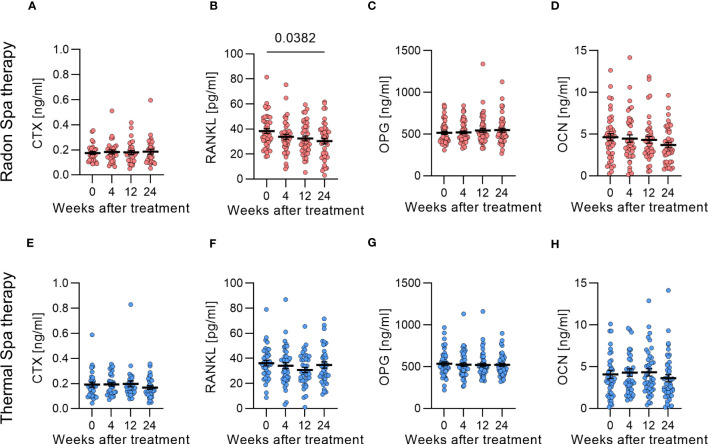
RANKL was significantly decreased only after radon spa treatment. No changes in CTX concentration after radon **(A)** and thermal spa treatment **(B)** were observed (N=29-30). RANKL was significantly decreased after radon **(C)**, but not after thermal spa treatment **(D)** (N=37-43). The bone formation markers OPG **(E, F)** (N=46-52) and OCN **(G, H)** (N=41-43) were not changed after radon **(E, G)** as well as after thermal spa **(F, H)** treatment. Significances were tested with Kruskal-Wallis test. Error bars are reported as mean ± SEM.

To show whether radon or thermal spa treatment leads to a systemic effect that could be the basis of the observed impact on osteoclastogenesis, we investigated bone resorption and bone formation markers in serum or plasma of the patients (**Figure 4**). As no carry over effect was identified, the data for the two groups, i.e. before or after cross-over, that had undergone the same treatment were pooled (see **Figure 1**). First, we examined the CTX concentration in the plasma of patients, but neither radon (**Figure 4A**) nor thermal (**Figure 4B**) spa treatment showed any influence. In contrast, compared to initial values before treatment, the RANKL concentration was reduced after radon spa treatment, and became significant 24 weeks after treatment (**Figure 4C**). Of note, the RANKL concentration was not changed after thermal spa treatment (**Figure 4D**). The results for the markers of bone formation showed no appreciable changes in the concentration of OPG (**Figures 4E, F**) and OCN (**Figures 4G, H**) after radon and thermal spa treatment compared to before treatment. We also determined the RANKL/OPG ratio, a well-known marker for bone metabolism. However, we did not detect any significant change after either radon or thermal spa treatment. There is only a trend towards a decrease after radon spa therapy, whereas it remains the same after thermal spa therapy (**Supplementary Figure 3**).

In summary, we observed an effect of radon and thermal spa treatment in the fraction of mOCs and resorbed area on bone slices (**Figure 3**), which was reflected after radon treatment on a systemic level for RANKL (**Figure 4C**).

### 3.3 No changes of MMP3 and MMP9 after radon and thermal spa treatment

MMPs are able to digest ECM. To find out whether radon and/or thermal spa treatment have an effect on MMP3 and MMP9, we examined the respective concentrations in serum of the patients’ blood before, 4, 12 and 24 weeks after treatment (**Supplementary Figure 1**). As no carry over effect was identified, the data for the respective series of the two groups that had undergone the same treatment were pooled (see **Figure 1**). The concentration of MMP3 in serum of patients’ blood was not changed at any time point after radon (**Supplementary Figure 1A**) and thermal (**Supplementary Figure 1B**) spa treatment. The same accounts for MMP9 (**Supplementary Figure 1C, D**).

In summary, at least on a systemic level, we did not observe an influence of both treatments on MMP3 and MMP9.

### 3.4 Adipokine levels are not altered after radon or thermal spa treatment

An association between OA and modified serum levels of adipokines has already been reported (43). Previously, we found that the adipokine visfatin was significantly reduced 12 weeks after radon spa treatment (15). To confirm this result in more patients we measured the concentration of the adipokines leptin and visfatin in serum of patients of the RAD-ON02 trial. As no carry over effect was identified, the data for the respective therapies of the two groups that had undergone the same treatment were pooled (see **Figure 1**). As shown in **Supplementary Figure 2**, trends but no significant changes were observed. The level of leptin was slightly increased after radon spa treatment, but slightly decreased after thermal spa treatment compared to the initial values before treatment, both changes not significant though (**Supplementary Figure 2A, B**). The visfatin concentration was slightly increased for both treatments 24 weeks after compared to before treatment (**Supplementary Figure 2C, D**).

### 3.5 Frequencies of Treg cells are significantly increased after radon spa treatment

**Figure 5:**
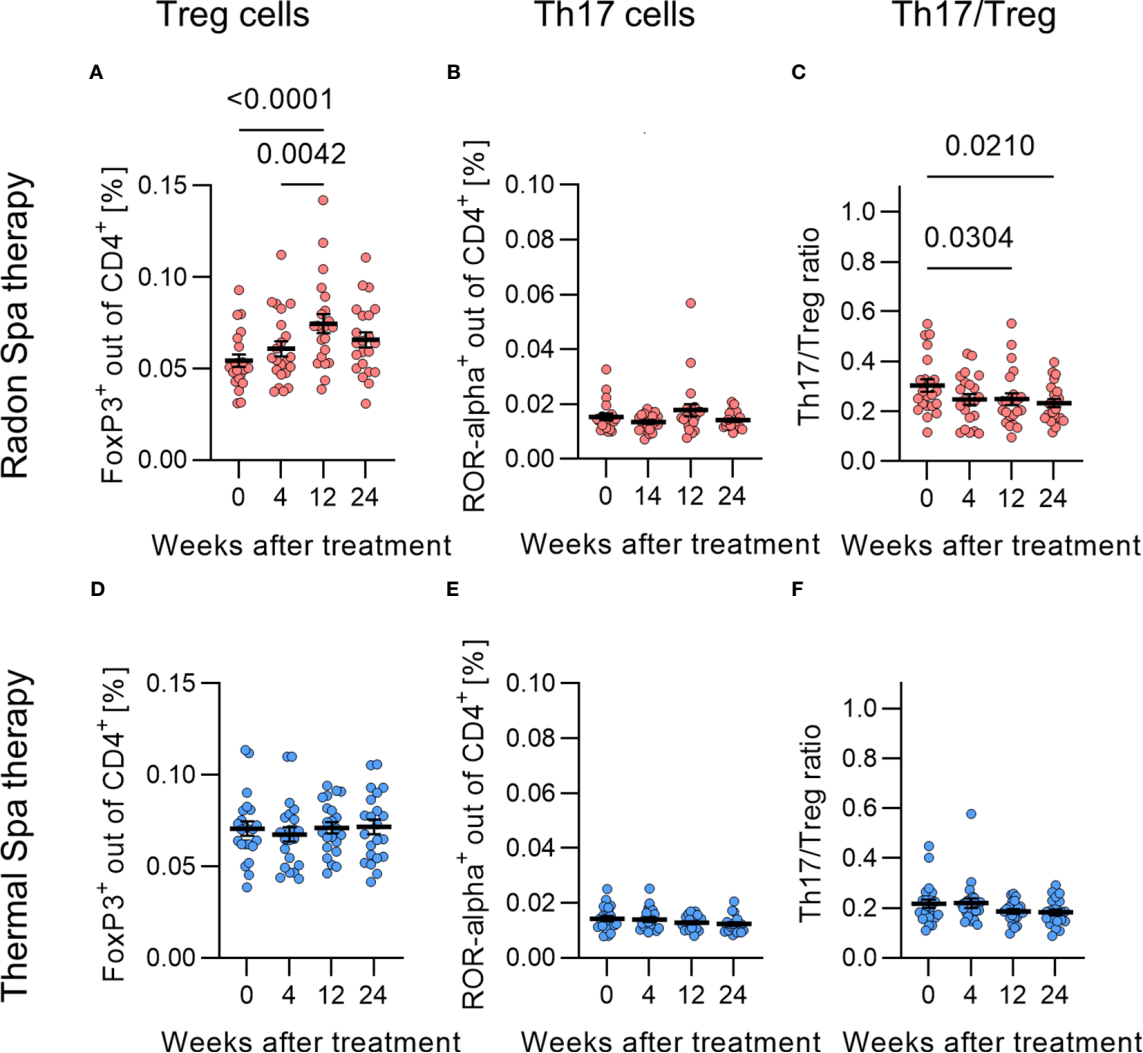
Treg cell population was significantly increased and Th17/Treg ratio significantly decreased after radon spa treatment. The fraction of Treg cells (CD4^+^ FoxP3^+^ cells) was significantly increased after Radon **(A)**, but not after thermal spa treatment **(B)**. No changes in the fraction of Th17 cells (CD4^+^ ROR- α^+^) were observed after radon **(C)** and thermal spa treatment **(D)**. In contrast, the Th17/Treg ratio was significantly reduced after radon **(E)**, but not after thermal spa treatment **(F)**. Significances were tested with RM one-way ANOVA for normal distributed data and Friedman test for non-normal distributed data. Error bars are reported as mean ± SEM (N=22).

A clear indicator of systemic immune effects with a potential direct relation to osteoclastogenesis is the ratio of Treg and Th17 cells (44). Therefore, we determined the frequency of Treg and Th17 cells in patients’ blood. Peripheral lymphocytes were isolated before, and 4, 12 and 24 weeks after treatment. As can be inferred from **Figure 5**, the fraction of Treg cells (CD4^+^FoxP3^+^) was significantly increased by 1.5-fold 12 weeks after radon spa treatment compared to before treatment (**Figure 5A**). In contrast, we could not detect any changes of the fraction of Treg cells after a thermal spa treatment (**Figure 5B**). For both treatments, the frequency of Th17 cells (CD4^+^ROR- α ^+^) was not changed (**Figures 5C, D**). Considering the ratio of Th17 to Treg cells (Th17/Treg), a significant decrease 12 and 24 weeks after radon spa treatment was detected in comparison to the initial time point (**Figure 5E**), but no changes have been found after thermal spa treatment (**Figure 5F**).

Taken together, we detected a significant impact of radon as well as thermal spa treatment on bone resorption and fraction of mOC. However, the effects were more pronounced after radon exposure for the RANKL concentration and Th17/Treg cells, which were related to the interaction between immune system and bone metabolism. In contrast, adipokines, matrix metalloproteinases, bone formation (OPG, OCN) and bone resorption (CTX) were not affected by radon or thermal spa treatment.

## 4 Discussion

The aim of this study was to investigate the effect of radon spa treatment on osteoclastogenesis and to compare the observed effects with thermal spa treatment. So far, previous studies have shown, that MSD patients show long-lasting pain reduction and a decrease of bone erosion markers such as CTX after radon spa treatment (9, 12, 15, 45, 46). One previous study showed that both radon and warm water bath applications lead to a reduction in pain in RA patients, but radon induced modifications persisted longer (11).

Based on this, our hypothesis was that radon as well as thermal spa treatments inhibit the differentiation and bone resorbing activity of OCs, which in turn leads to pain relief. More pronounced effects are expected after radon spa treatment. To investigate this in patients that underwent either radon or thermal spa treatment in two consecutive years (with a crossover of the treatment modalities in the second year), we performed an *ex vivo* study on monocytes isolated from patients’ blood before and at different time points after treatment (**Figure 1**). The monocytes were cultivated on bone slices in the presence of growth factors (RANKL, M-CSF) for 21 days. We observed a reduced fraction of mOCs after both treatments, but the level of significance was higher after radon compared to thermal spa treatment. This was also reflected in a lower resorption activity of mOCs on the bone slices after radon compared to thermal spa treatment (**Figure 3**).

To the best of our knowledge, this is the first study assessing osteoclastogenesis on bone slices after radon therapy *ex vivo*. Comparable investigations were reported in monocytes from healthy donors that were *ex vivo* cultivated after X-ray exposure (38) from our group, and by others in an arthritic mouse model after local X-ray exposure (47).

The comparison between radon and LDRT treatment is very complex, although both are considered low-dose irradiations. Radon spa is applied as a whole-body exposure, whereas LDRT is only applied locally on the affected joint. Also, the physical doses delivered are in different orders of magnitudes, i.e. 10 x 1-10 µGy for radon spa (48–50) versus 10 x 0.5 Gy for LDRT (6). In addition, both treatment modalities differ in their physical properties. X-ray is classified as sparsely-ionizing radiation, whereas radon is classified as densely-ionizing radiation due to the high dose contribution of α-particles in the decay process of radon. In addition X-ray is purely external irradiation whereas radon enters the body via inhalation and attachment to the epithelial surfaces (further details in Maier at al., 2021) (48).

However, both LDRT and radon treatment are used to treat MSDs and lead to significant pain reduction (8, 10, 51). In accordance with the significant decrease in mOCs after radon spa treatment, Deloch et al. showed the same effect after local X-ray irradiation in a transgenic arthritic mouse model (*h*TNF-α tg mice). They demonstrated a significant decrease of mOCs and also a lower bone resorbing activity (47). Consistently, the same authors observed that the bone resorbing abilities of healthy osteoclasts, differentiated from bone-marrow cells from C57Bl/6 animals and irradiated *ex vivo*, was slightly reduced by X-ray irradiation (52).

However, for X-ray exposure our own previously reported results obtained *in vitro* showed that for human, *ex vivo* irradiated monocytes that were cultivated on bone slices, the fraction of mOCs, resorption activity and CTX release into the medium were significantly reduced. As one contributing mechanism we identified the translocation of Nuclear factor of activated T cells cytoplasmic-1 (NFATc1) into the nucleus (38). This comparison does not take into account the difference in radiation quality and exposure situation, neither between *in vitro* and *in vivo*, but seems interesting because this *in vitro* X-ray study reveals similar radiation responses of OC as identified in the RAD-ON 02 study.

In the here presented results obtained in the frame of the RAD-ON02 study, we quantified in addition to the resorption activity and the fraction of mOCs, markers for bone resorption and formation in serum and plasma of MSD patients. We observed that RANKL was significantly decreased after radon spa treatment (**Figures 4C**), and, in line with the role of RANKL in osteoclastogenesis (17), a lower resorbing activity of mOCs (**Figure 3**). OPG, an antagonist of RANKL, was not changed, irrespective of the treatment (**Figures 4E, F**).

These latter results are not in agreement with the respective results obtained in the preceding RAD-ON01 trial, where RANKL levels were not modified and the OPG concentration was decreased after radon spa treatment (15). A comparison of the patients enrolled in the RAD-ON01 and the RAD-ON02 study and then randomly selected for the deeper analysis of the selected markers, show differences with respect to the specific types of MSD diseases of the patients (15). In the RAD-ON01 study, 40% of the patients compared to only 10% of the patients recruited in the RAD-ON02 study suffered from degenerative diseases of the spine. Only 16% of the RAD-ON01 patients were diagnosed multiple indications (more than one joint affected by the degenerative disease) in contrast to 86% of the RAD-ON02 patients. Thus, both cohorts are not comparable and we assume that the different effects are due to the heterogeneity of the patient cohorts in both studies (15).

The minor comparability of cohorts is a general problem when comparing with other studies. Further indication for a decrease of RANKL after treatment in a radon spa was reported for persons, which were healthy and at the risk age for developing osteoporosis. Moreover, in parallel to the spa treatment, they underwent physical activity. It is clear, that the inflammatory status of the individuals (healthy or suffering from MSD) and the physical activity in this study have a major influence on bone metabolism (53).

In line with our own results, for OA patients a significant reduction of RANKL and no change of OPG after radon treatments in gallery was observed compared to before treatment, whereas in RA patients in the same study, a significant increase in OPG concentration was observed after radon spa treatment (54). This is in line with results from preclinical low-dose studies (47). The corresponding systemic markers for bone resorption are the collagen fragments measured in the plasma of the patients, i.e. CTX (**Figure 4**). For the RAD-ON02 cohort, the CTX concentration was unchanged at all time points both after radon and thermal spa treatment compared to before therapy (**Figures 4A, B**). On the contrary, the CTX concentrations in plasma of RAD-ON01 patients showed a significant decrease starting from 12 weeks after radon spa treatment compared to before treatment (15). In addition to the above-mentioned differences in the cohorts of both studies, it is also likely that systemic markers indicating local processes such as bone erosion are not robust enough to yield consistent results.

However, CTX is mostly measured in urine. Gaisberger et al. measured the urinary CTX level in OA patients, which slightly declined both with and without radon treatment, indicating slight changes of CTX, independent of radon exposure. Both groups of patients received the same basis therapies (massage, physical training). Unfortunately, the study was not blinded and performed in a small cohort of patients, therefore the significance of the results is limited (55).

We also investigated markers for bone formation in the plasma of patients in this study. The OCN concentration did not change after both treatments (**Figure 4**). The mean concentration of OCN before therapy was 4.3 ng/mL, which corresponds to other measurements in healthy individuals, for example by Stracke et al. who measured a mean value of 4.1 ng/ml OCN (56). This indicates normal bone formation and no modification after both treatments, which confirms the results obtained in the RAD-ON01 study for radon spa treatment (15). Interestingly, Winklmayr et al. have reported an increase in the OCN concentration in plasma of healthy donors at the risk age for developing osteoporosis in the radon as well as in the placebo group (53).

To investigate whether radon treatment has an influence on cartilage-degrading enzymes such as MMP, we examined MMP3 and MMP9 in the serum of the patients. Radon and thermal spa treatment showed no effect on MPP3 and MPP9 concentrations in this patient cohort (**Supplementary Figure 1**). Notably, the concentrations of MMP3 and MMP9 for the RAD-ON02 cohort before therapy are within the range of the values of healthy donors in serum (MMP3: 15.37 ng/ml, MMP9: 329 pg/ml) (57), indicating no major cartilage destruction before treatment.

As already mentioned, the solubility of radon in fat is higher than in water (37). In addition, adipokines released by fat cells are known to show an increased systemic level in OA patients (30, 31). Hence, radon could modulate the release of adipokines in fatty tissue. In this study, we determined both visfatin and leptin concentration in serum of patients, which were not changed after both radon and thermal spa treatment (**Supplementary Figure 2**). For leptin, this is in line with the results obtained in the RAD-ON01 study (15). We evaluated the body-mass-index of the patients before and during each follow-up after therapy, also to rule out an anti-correlated impact of changes in BMI, potentially masking treatment induced changes of the leptin levels. The weight of the patients underwent slight changes, not translating into a changed BMI, and thus not explaining the unchanged levels of leptin in this study. The visfatin concentration in the serum of patients of the RAD-ON01 cohort was significantly reduced from 12 weeks after radon spa treatment (15). We assume that for adipokines, among the differences in the cohorts of patients an important parameter is the weight of the patients, which has a major influence on the amount of adipokines released. This impact cannot be taken into consideration in the case of the RAD-ON01 study, because the weight of the patients was not part of the medical follow-up. Notably, among the patients of the RAD-ON02 study, a majority (52%) of them are overweight and even 22% obese. In line with other parameters measured, the visfatin concentration in the serum of the patients enrolled in both studies (RAD-ON02 and RAD-ON01) are within the range of healthy donors (visfatin 2-3 ng/ml) (15, 58).

A complex interaction between bone and immune cells results in the regulation of bone resorption and formation (59). Thus, the abundance of T cell subtypes in the peripheral blood with opposed effects, i.e. Treg and Th17 cells, correlates with bone resorption markers (44). In the patients cohort of the RAD-ON02 and RAD-ON01 study (15), we showed that the population of Treg cells significantly increased after radon application. In the RAD-ON02 study it turned out that this was not the case after thermal spa treatments, suggesting that this is a radon-mediated effect. Notably, the population of Th17 cells remained unchanged after both therapies, resulting in a significant decrease in the Th17/Treg ratio after radon treatment (**Figure 5**). This finding could be related to the reduced terminal differentiation and activity of mOCs, as the increased population of Treg cells may reduce the differentiation into mature OCs (60, 61). The additional role of immune effects after radon spa treatment could be responsible for the more pronounced effects related to bone erosion for radon spa compared to thermal spa treatment.

In conclusion, we could demonstrate that radon spa treatment has an effect on the fraction and resorbing activity of mOCs, the RANKL concentration in serum, and on the T cell subtypes, which are abundant in the peripheral blood, i.e. the fraction of Treg cells that inhibits immune activity and osteoclastogenesis. However, we could not confirm previous results on other markers of bone resorption (CTX) and adipokines (visfatin), indicating the importance of a well-characterized patient cohort. These markers and markers of cartilage destruction, i.e. MMP3 and 9, were in the MSD cohorts investigated at levels of healthy individuals and therefore most likely only slightly changed, independent of radon exposure, or not changed at all.

Taken together, our results showed that the observed influence on bone destruction and immune suppression are not based on radon exposure alone, but are probably more pronounced for radon spa treatment. This highlight the importance of a placebo control in a trial. In order to investigate the osteo-immunological influence of radon therapy on MSD patients, the patient collective should be defined more precisely (disease status) in future studies.

## Data availability statement

The raw data supporting the conclusions of this article will be made available by the authors, without undue reservation.

## Ethics statement

The studies involving humans were approved by EudraCT Nr. 2016-002085-31 DRKS-ID DRKS00016019. The studies were conducted in accordance with the local legislation and institutional requirements. The participants provided their written informed consent to participate in this study.

## Author contributions

DE: Data curation, Formal Analysis, Investigation, Methodology, Project administration, Resources, Software, Validation, Visualization, Writing – original draft, Writing – review & editing. MEv: Data curation, Formal Analysis, Investigation, Methodology, Visualization, Writing – original draft, Writing – review & editing. JS: Data curation, Formal Analysis, Investigation, Methodology, Visualization, Writing – original draft, Writing – review & editing. MI: Data curation, Formal Analysis, Investigation, Methodology, Visualization, Writing – original draft, Writing – review & editing. MEr: Data curation, Formal Analysis, Investigation, Methodology, Visualization, Writing – original draft, Writing – review & editing. LD: Data curation, Formal Analysis, Investigation, Methodology, Visualization, Writing – original draft, Writing – review & editing. FR: Conceptualization, Data curation, Formal Analysis, Investigation, Methodology, Project administration, Resources, Software, Validation, Visualization, Writing – original draft, Writing – review & editing. A-JD: Conceptualization, Project administration, Validation, Writing – review & editing. UG: Project administration, Supervision, Validation, Writing – review & editing, Conceptualization, Funding acquisition. BF: Conceptualization, Funding acquisition, Project administration, Supervision, Validation, Writing – review & editing. CF: Conceptualization, Data curation, Formal Analysis, Funding acquisition, Investigation, Methodology, Project administration, Resources, Software, Supervision, Validation, Visualization, Writing – original draft, Writing – review & editing.
