# Supplementary material for: Osteo-immunological impact of radon spa treatment: due to radon or spa alone? Results from the prospective, thermal bath placebo-controlled RAD-ON02 trial
Source: Front Immunol. 2024 Jan 16;14:1284609. doi: 10.3389/fimmu.2023.1284609 (PMC10824901; doi:10.3389/fimmu.2023.1284609)
Supplement: Supplementary file 1 [file DataSheet_1.docx]

Osteo-immunological impact of radon spa treatment: Due to radon or spa alone?

Results from the prospective, thermal bath placebo-controlled
RAD-ON02 trial

Denise Eckert^1^, Megi Evic^1^, Jasmin Schang^1^, Maike Isbruch^1^, Melissa Er^1^, Lea Dörrschuck^1^, Felicitas Rapp^1+^, Anna-Jasmina Donaubauer^2^, Udo S. Gaipl^2^, Benjamin Frey^2^ and Claudia Fournier^1*^

^1^Department of Biophysics, GSI Helmholtzzentrum für Schwerionenforschung, Darmstadt, Germany

^2^Translational Radiobiology, Department of Radiation Oncology, Universitätsklinikum Erlangen, Friedrich-Alexander-Universität Erlangen-Nürnberg (FAU), Erlangen, Germany

^+^Present address: Department of Hematology and Oncology, Medical Faculty Mannheim, Heidelberg University, Mannheim, Germany

*** Correspondence:**Prof. Dr. Claudia Fournier
c.fournier@gsi.de

Supplemental

Table S1 Inclusion and exclusion criteria for inclusion into the RAD-ON02 study

| **Inclusion criteria** | **Exclusion criteria** |
| --- | --- |
| - Age at least 18 years, up to 75 years - Chronic degenerative spinal and joint complaints - Duration of pain for at least 1 year - Pain intensity VAS ≥4 - Effective contraception - Accessibility of patients (geographical proximity) for treatment and follow-up - Willingness of patients to cooperate - Completed patient education and provided written informed consent - No participation in other studies (3 months before and) during participation | - Pregnant or breastfeeding women - Those capable of childbearing or procreation who are unwilling or unable to use consistent contraceptive measures during therapy - Heart failure > NYHA II or EF<40%. - Uncontrollable hypertension > 180/90 mmHg - Manifest hyperthyroidism that cannot be controlled with medication - Acute inflammatory or consumptive processes - Successful radon treatment within 9 months prior to study entry - Successful radiation therapy in the course of tumor disease - Persistent drug, medication or alcohol abuse - Patients unable or unwilling to comply with protocol and receive treatment - Unwillingness to store and share personal medical records as part of the protocol - Concurrent participation in another clinical trial |

**Table S2 Detailed** **Characteristics of the patients (group1 and group2)**

|  |  | Group 1 | Group 2 |  |
| --- | --- | --- | --- | --- |
| Total number | | 29 | 29 | patients |
| Age at start | Mean | 61 | 59 | Years |
|  | Range | 47-68 | 40-73 | Years |
| Gender | Male | 12 | 8 | patients |
|  | Female | 17 | 21 | patients |
| BMI | Normal (<25) | 4 (13.8 %) | 10 (34.5 %) | patients |
|  | Overweight (25-30) | 19 (65.5 %) | 13 (44.8 %) | patients |
|  | Obese (>30) | 6 (20.7 %) | 6 (20.7 %) | patients |
| Indications | Big Joints | 2 (6.9 %) | 2 (6.9 %) | patients |
|  | Spine | 4 (13.8 %) | 3 (10.3 %) | patients |
|  | Multiple Indications | 23 (78.3 %) | 24 (82.8 %) | patients |


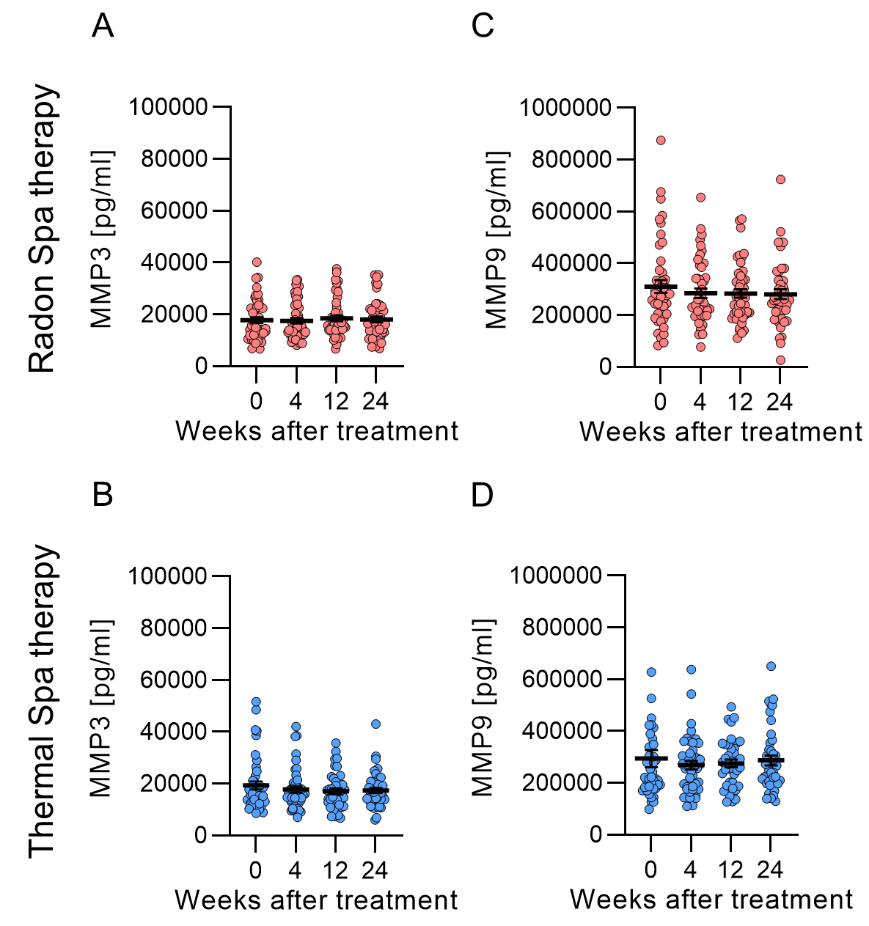


*Figure S1 Both radon and thermal spa treatment had no effect on the matrix metalloproteinases (MMP3 and MMP9). No influence on MMP3 (A,B) and MMP9 (C,D) were detected after radon (A,C) and thermal (B,D) spa treatment. Significances were tested with Kruskal-Wallis test. No significances have been identified. Error bars are reported as mean ± SEM (N=46-47).*


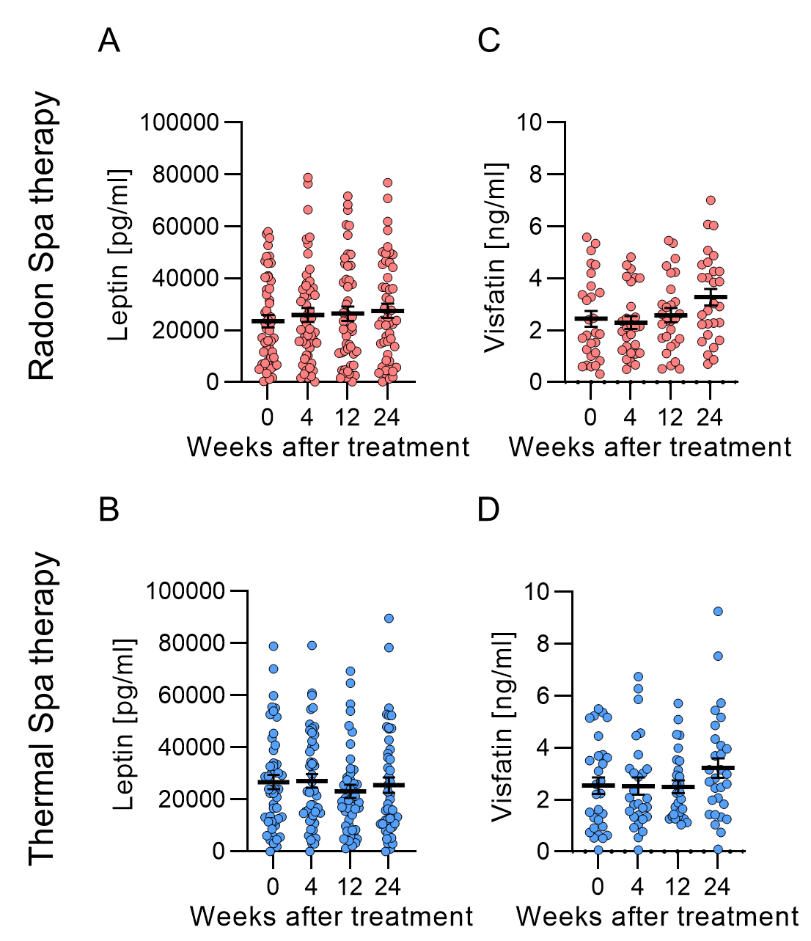


*Figure S2 Both radon and thermal spa treatment had no significant effect on adipokines (Visfatin and Leptin). No influence on Leptin (A,B) and Visfatin (C,D) were shown after radon (A,C) and thermal (B,D) spa treatment. Significances were tested with Kruskal-Wallis test. No significances have been identified. Error bars are reported as mean ± SEM (N=46-47).*


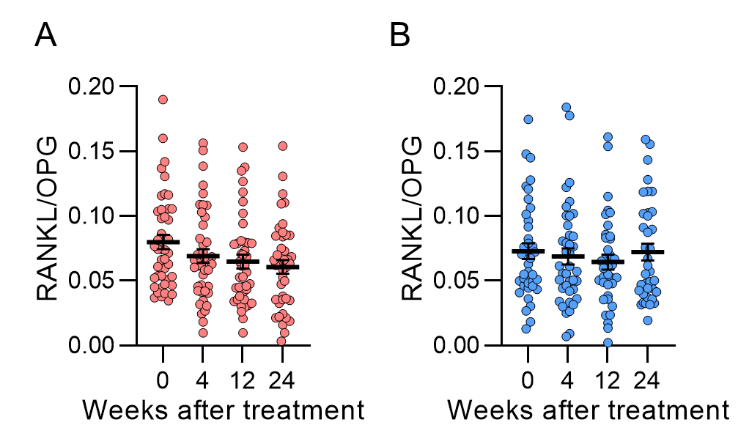


*Figure S3 Both radon and thermal spa treatment had no significant effect on RANKL/OPG ratio. No influence on RANKL/OPG were shown after radon (A) and thermal (B) spa treatment. Significances were tested with Kruskal-Wallis test. No significances have been identified. Error bars are reported as mean ± SEM (N=43-45).*
